# The sex impact on the technological and chemical characteristics of meat derived from the Polish native chicken breed

**DOI:** 10.1038/s41598-023-33430-6

**Published:** 2023-04-21

**Authors:** Kornel Kasperek, Kamil Drabik, Weronika Sofińska-Chmiel, Małgorzata Karwowska, Grzegorz Zięba, Justyna Batkowska

**Affiliations:** 1 Institute of Biological Basis of Animal Production, University of Life Sciences in Lublin, 13 Akademicka St., 20-950 Lublin, Poland; 2 Analytical Laboratory, Institute of Chemical Sciences, Faculty of Chemistry, Maria Curie Skłodowska University, 3 Maria Curie Skłodowska Sq., 20-031 Lublin, Poland; 3 Department of Meat Technology and Food Quality, University of Life Sciences in Lublin, 8 Skromna St., 20-704 Lublin, Poland

**Keywords:** Metabolism, Hormones, Lipids, Biochemistry, Imaging

## Abstract

The aim of the study was to evaluate the birds’ sex as well as the caponisation influence on the technological traits of obtained meat, fatty acids profile as well as main chemical compounds distribution. In this study, 40 hens, roosters and green-legged partridge capons each were used (4 replications each). At 24 weeks of age, 2 birds from each replication subgroup were selected and slaughtered. During dissection analysis, pectoral and femoral muscles were sampled. Acidity, colour, tenderness, water holding capacity, drip and cooking loss were determined in the obtained material. The fatty acid profile of the meat was also determined, as well as the distribution of components in the pressed meat samples using FTIR spectroscopy. Statistically significant differences in the colour lightness index (L*) of the breast muscles were recorded, with cockerel meat being the darkest and capon meat the lightest. The greatest natural water loss was recorded in the hens' pectoral muscle; cooking loss was also the greatest in these samples. Roosters showed significantly the lowest content of monounsaturated fatty acids, at the same time the highest proportion of the n3 fatty acids group was found in capons. Irrespective of muscle, meat from roosters showed the highest susceptibility to oxidation (PI index). The variation in the chemical composition of the meat was confirmed by FTIR mapping.

## Introduction

Poultry production is one of the largest livestock sectors. By providing relatively cheap and readily available animal protein, commodity poultry production is one of the most significant food sources in both developed and developing countries.

Although at present the majority of poultry meat is obtained from fast-growing chicken broilers, consumer trends indicating a search for products of extensive systems and the sourcing of raw material from birds with reduced growth rates are becoming apparent^1^.

Furthermore, it has been shown that meat from free-range birds, compared to typical broiler chickens, differs in terms of quality, chemical composition or consumer acceptability^2^. Napolitano et al.^3^ showed that meat from free-range birds can be perceived as less tender and less juicy compared to commercial broiler chickens. At the same time, it should be noted that often, in addition to the already-known meat quality characteristics, consumers' purchasing choices are also influenced by a lack of knowledge or habits^4^. From this perspective, it seems reasonable to source raw materials from native breeds as potentially familiar and traditional for particular local markets.

Consequently, many research works focusing on caponisation use native breeds as test material. In Poland, these have primarily been Green Partridge (GP)^5,6^, although other breeds such as Castellana Negra^7^ or Nara chickens^8^ have also undergone similar treatments. Regardless of the test material used, the aim of caponing is to obtain meat with improved flavour and altered fat content compared to cockerels and to obtain the desired meat texture through changes in muscle fibre diameter^5^.

One reason for the changes in meat quality is the disturbance in androgen levels resulting from the surgery, as they play an important role in the lipid metabolism of birds^9^. However, almost all works investigating the effect of caponisation on meat quality focus exclusively on the cock vs. capon comparison system. The available literature lacks data on capon, cockerel and hen comparisons. Although available reports compare birds (males and females) undergoing castration^10^, there are very few full comparisons within a single breed. It seems that a full comparison includes both males, and females and can provide valuable comparative material both from a production and strictly biological point of view.

Classically, technological parameters (i.e. colour, tenderness) and chemical composition including fatty acid profile are assessed among the basic meat evaluation parameters. This allows a broad assessment of raw material quality but is labour-intensive. Therefore, other analytical techniques such as Fourier-transform infrared (FTIR) spectroscopy are increasingly being used, which, due to its high accuracy and lower labour-intensity of the analysis process, is readily used in the analysis of quality, adulteration or safety of meat raw materials^11^.

The aim of the study was to evaluate the birds’ sex as well as the caponisation influence on the technological traits of obtained meat (pH, colour coordinates, drip and cooking loss, water holding capacity and shear force), fatty acids profile as well as main chemical compounds distribution.

## Results

Analysing the technological characteristics of the birds' muscles according to their sex (Table 1), it was found that the highest pH of the pectoral muscle 60 min after slaughter was characterised by hens, with the lowest values of this characteristic in the muscles of roosters. In the case of the thigh muscle for the same time, a significantly lower pH value was found for capons, while hen and cockerel muscles did not differ significantly for this trait.

**Table 1:** Technological characteristic of muscles.

Item	Breast muscle						Thigh muscle					
	Rooster		Hen		Capon		Rooster		Hen		Capon	
	Mean	SD	Mean	SD	Mean	SD	Mean	SD	Mean	SD	Mean	SD
pH_15_	5.89	0.325	5.96	0.352	5.74	0.246	5.600	0.180	5.74	0.182	5.75	0.243
pH_60_	5.57^a^	0.060	5.68^b^	0.128	5.63^ab^	0.131	5.73^b^	0.116	5.71^b^	0.189	5.54^a^	0.191
pH_24h_	5.44	0.097	5.45	0.149	5.41	0.146	5.63	0.119	5.71	0.156	5.70	0.093
*Colour coordinates*												
Raw												
L*	57.08	6.272	60.13	2.592	60.01	6.055	42.36	5.289	45.20	4.064	45.75	5.040
a*	-0.339	1.339	-1.006	0.752	0.141	3.316	12.71	3.526	12.67	1.618	12.14	3.285
b*	10.34	2.333	12.55	1.234	12.13	3.650	9.85	3.243	10.71	3.498	10.91	3.600
C	10.44	2.272	12.62	1.183	12.41	4.118	16.19	4.351	16.72	2.718	16.43	4.449
h	92.23	8.622	94.77	4.004	92.49	10.675	37.25	7.811	39.27	10.939	40.41	9.296
Cooked												
L*	84.12^a^	5.461	85.97^ab^	1.895	87.31^b^	1.195	67.62	4.361	70.60	8.557	72.54	7.313
a*	1.104	1.112	0.571	1.118	0.376	0.282	5.39	1.821	4.40	2.298	4.06	1.376
b*	14.65^b^	1.726	14.73^b^	0.893	13.42^a^	1.113	17.77	1.426	16.77	2.092	17.09	1.595
C	14.72^b^	1.820	14.78^b^	0.977	13.43^a^	1.117	18.64	1.617	17.44	2.392	17.59	1.822
h	85.99	3.489	87.96	3.892	88.44	1.180	73.32	5.077	75.90	6.826	76.94	3.556
ΔE	27.75	6.465	26.06	2.952	27.90	5.176	27.90	2.808	27.88	7.612	29.68	7.851
Drip loss (%)	2.16^a^	0.619	2.84^b^	0.723	2.12^a^	0.510	2.16	0.625	2.08	1.029	2.19	0.541
Cooking loss (%)	28.86	8.253	29.21	10.942	26.54	6.358	28.81	8.321	27.71	13.724	29.19	7.200
Water holding capacity (cm^2^)	44.01	10.238	37.25	9.379	45.567	12.987	41.37	7.410	40.84	8.312	36.43	7.628
Shear force (N)	26.93	7.839	28.24	9.148	27.384	7.192	43.90	11.826	37.27	7.952	44.17	10.615

ΔE The change of meat colour coordinates after thermal processing, ^a,b^Mean values in the row (among particular muscle) differ significantly at *p* ≤ 0.05; *SD* standard deviation.

Despite the lack of significant differences in the colour of raw breast muscle in all groups, significant differences were observed for heat-treated muscle samples. It was found that after the thermal preparation the highest meat lightness (L*) was characteristic of capons with the lowest values of this trait recorded for meat from roosters. In the case of this parameter, hen breast muscles did not differ significantly from the other groups of birds.

Other trends were observed in the context of yellow colour saturation (b*) and chromaticity (C) of the pectoral muscle samples analysed. The lowest values of the two traits were found in capons, with no significant differences between muscles obtained from GP hens and roosters.

Significant differences were also found in the drip loss of the pectoral muscles depending on the group of birds. The hen muscles had the highest loss, with lower values recorded for capons and roosters. For the other technological traits (cooking loss, WHC, shear force), no significant differences were found between groups.

Analysis of the fatty acid profile (Tables 2, 3) showed a slight variation within the analysed groups of birds. In the context of the pectoral muscle, the values of margaric and behenic fatty acids were found to be significantly highest in cockerel muscle samples, with the lowest values recorded in hen muscle samples. At the same time, the pectoral muscle of capons did not differ in this respect from the other groups tested. Within unsaturated fatty acids, only palmitoleic acid differentiated the pectoral muscle samples. A considerably higher proportion of this acid was found in hen meat, the lowest in cocks’ muscles.

**Table 2:** Saturated fatty acid profile of analysed muscles.

Item (%)	Breast muscle						Thigh muscle					
	Rooster		Hen		Capon		Rooster		Hen		Capon	
	Mean	SD	Mean	SD	Mean	SD	Mean	SD	Mean	SD	Mean	SD
Saturated fatty acids (SFA)												
C6:0	0.070	0.058	0.020	–	0.030	–	0.021	0.015	0.015	0.009	tr	–
C8:0	0.108	0.085	0.070	0.057	0.070	0.071	0.035	0.021	0.040	0.014	0.080	–
C10:0	0.208	0.231	0.100	–	0.047	0.038	0.060	0.057	0.040	–	0.020	–
C12:0	0.318	0.369	0.156	0.282	0.152	0.268	0.054	0.051	0.032	0.011	0.024	0.005
C14:0	1.174	0.886	0.548	0.127	0.592	0.126	0.496	0.114	0.592	0.209	0.452	0.228
C15:0	0.192	0.109	0.094	0.011	0.108	0.015	0.122	0.011	0.102	0.016	0.118	0.011
C16:0	20.76	2.010	21.51	1.275	20.75	1.154	18.29	0.697	20.90	2.920	20.12	1.644
C17:0	0.202^b^	0.041	0.148^a^	0.021	0.176^ab^	0.019	0.208^b^	0.015	0.166^a^	0.032	0.160^a^	0.021
C18:0	7.356	0.842	5.878	0.890	7.606	2.324	8.322^b^	0.873	5.636^a^	1.686	5.322^a^	0.714
C20:0	0.106	0.024	0.053	0.013	0.120	0.069	0.148^b^	0.054	0.074^a^	0.023	0.072^a^	0.016
C22:0	0.088^b^	0.017	0.035^a^	0.021	0.048^ab^	0.018	0.070	0.016	0.032	0.013	0.048	0.052
C23:0	0.080	–	0.410	–	tr	–	tr	–	0.050	–	0.070	–
C24:0	0.058	0.021	0.030	-	0.025	0.007	0.042	0.011	0.020	–	tr	–

^a,b^Mean values in the row (among particular muscle) differ significantly at p ≤ 0.05; *SD* standard deviation, C 6:0—caproic acid, C8:0—caprylic acid, C10:0—capric acid, C12:0—lauric acid, C14:0—myristic acid, C15:0—pentadecylic acid, C16:0- palmitic acid, C17:0—margaric acid, C18:0—stearic acid, C20:0—arachidic acid, C22:0—behenic acid, C23:0—tricosanoic acid, C24:0—lignoceric acid.

**Table 3:** Monounsaturated and polyunsaturated fatty acid profile of analysed muscles.

Item (%)	Breast muscle						Thigh muscle					
	Rooster		Hen		Capon		Rooster		Hen		Capon	
	Mean	SD	Mean	SD	Mean	SD	Mean	SD	Mean	SD	Mean	SD
Monounsaturated fatty acids (MUFA)												
C14:1 n5	0.120	0.072	0.106	0.045	0.094	0.027	0.058^a^	0.015	0.092^ab^	0.016	0.118^b^	0.030
C15:1 n5	tr		tr		tr		tr		0.030		0.020	
C16:1 n7	2.292^a^	0.311	3.234^b^	0.720	3.09^ab^	0.552	2.032^a^	0.418	4.038^b^	0.763	4.296^b^	0.931
C18:1 n9	30.74	1.985	34.46	5.485	33.85	1.977	32.16^a^	1.756	38.18^b^	1.551	37.00^b^	0.936
C20:1 n9	0.324	0.050	0.294	0.018	0.364	0.066	0.476	0.076	0.370	0.047	0.410	0.041
C22:1 n9	0.040		0.025	0.006	0.036	0.013	0.060	0.024	0.048	0.044	0.116	0.198
Polyunsaturated fatty acids (PUFA)												
C18:2 n6	23.88	3.500	22.00	0.646	23.79	2.509	28.39	2.69	22.08	5.643	24.20	4.144
C18:3 n6 γ	0.082	0.024	0.095	0.010	0.068	0.022	0.070	0.024	0.076	0.019	0.056	0.023
C18:3 n3 α	1.138	0.157	1.206	0.079	1.392	0.168	1.346	0.248	1.190	0.318	1.310	0.347
C20:2 n6	0.286	0.071	0.214	0.043	0.254	0.021	0.340^b^	0.048	0.208^a^	0.058	0.226^a^	0.036
C20:3 n6	0.174	0.067	0.140	0.049	0.138	0.053	0.126	0.025	0.104	0.030	0.088	0.035
C20:4 n6	2.044	0.728	1.072	0.596	1.200	0.594	1.614^b^	0.441	0.524^a^	0.180	0.500^a^	0.263
C20:3 n3	0.020	–	0.020	–	0.026	0.009	0.028	0.005	0.023	0.006	0.020	–
C20:5 n3	tr	–	0.020	–	0.040	–	0.020	–	0.027	0.012	0.130	–
C22:2 n6	0.194	0.196	0.049	0.016	0.068	0.033	0.224	0.246	0.058	0.011	0.062	0.023

^a,b^Mean values in the row (among particular muscle) differ significantly at *p* ≤ 0.05; *SD* standard deviation, C14:1—tetradecenoic acid, C15:1—pentadecenoic acid, C16:1—palmitoleic acid, C18:1—oleic acid, C20:1—gondoic acid, C22:1—nervonic acid, C18:2 n6—linoleic acid (LA), C18:3 n6- γ-linolenic acid (GLA), C18:3 n3- α—linolenic acid (ALA), C20:2n6—eicosadienoic acid, C20:3 n6- dihomo-γ-linolenic, C20:4 n6- arachidonic acid (AA), C20:5 n3—eicosapentaenoic acid, C22:2 n6- docosadienoic acid, tr- trace- content lower than 0.05%.

Noticeably more differences in the fatty acid profile were recorded for the thigh muscles. In the case of margaric, stearic and arachic acids, their significantly lower proportions were found in capons' muscles compared to roosters. At the same time, the differences between the thigh muscles of hens and cockerels were not significant. Among monounsaturated fatty acids, a significantly higher proportion of tetradecenoic acid and palmitoleic acid was observed in the thigh muscles of capons compared to those obtained from roosters (Table 3). At the same time, the values for the proportion of these fatty acids were similar in hens' and capons' meat. The inverse relationship in this respect concerns polyunsaturated fatty acids. Higher contents of eicosadeic and arachidonic acids were found in femoral muscle samples from roosters compared to hens and capons, for which no differences were observed.

Based on the proportion of particular fatty acids in the muscle samples, their indexes were calculated (Table 4). The highest MUFA content was found in hens' pectoral muscle, with the lowest values in roosters' pectoral muscle, while capons did not differ significantly from the other groups. A different trend was observed for the thigh muscles. Muscles obtained from roosters contained considerably the least MUFA, but no significant differences were observed between hens' and capons' muscles. The content of n3 polyunsaturated fatty acids varied between the birds' pectoral muscles according to their sex. It was found that the highest n3 content was observed in the pectoral muscles of capons, with the lowest values in roosters' pectoral muscles.

**Table 4:** Fatty acid indexes of analysed muscles.

Item (%)	Breast muscle						Thigh muscle					
	Rooster		Hen		Capon		Rooster		Hen		Capon	
	Mean	SD	Mean	SD	Mean	SD	Mean	SD	Mean	SD	Mean	SD
SFA	30.64	4.341	28.41	1.156	29.62	3.443	27.81	0.361	27.51	4.786	26.46	2.281
MUFA	33.50^a^	2.199	40.11^b^	3.998	37.43^ab^	2.414	34.78^a^	2.060	42.07^b^	2.678	41.86^b^	1.589
PUFA	27.81	3.200	24.90	1.070	26.95	2.532	32.12^b^	2.509	22.54^a^	5.268	26.48^ab^	4.687
n3	1.146^a^	0.163	1.230^ab^	0.092	1.430^b^	0.178	1.372	0.253	1.278	0.205	1.352	0.298
n6	26.66	3.057	23.67	1.120	25.52	2.427	30.75^b^	2.272	23.05^a^	5.877	25.13^ab^	4.396
n9	31.07	1.977	34.37	5.678	34.25	2.002	33.30^a^	2.781	37.93^b^	2.250	37.44^b^	0.951
PI	36.18^b^	2.578	30.43^a^	2.335	33.15^ab^	3.309	39.43^b^	2.358	28.84^a^	6.072	30.63^a^	5.334
AI	0.426	0.121	0.368	0.023	0.362	0.040	0.304	0.017	0.356	0.092	0.322	0.049
TI	0.768	0.177	0.786	0.053	0.762	0.171	0.604	0.050	0.864	0.393	0.718	0.239
DFA	68.66	3.506	70.89	2.444	71.99	2.216	75.23	1.290	72.44	4.989	73.67	2.867
HSFA	22.26	3.009	22.21	1.070	21.49	1.204	18.84	0.669	21.53	3.108	20.60	1.783
h:H	2.67	0.532	2.66	0.171	2.836	0.340	3.398	0.152	2.98	0.714	3.10	0.419

^a,b^Mean values in the row (among particular muscle) differ significantly at *p* ≤ 0.05; *SD* standard deviation, *SFA* saturated fatty acids, *UFA* unsaturated fatty acids, *PUFA* polyunsaturated fatty acids, *MUFA* monounsaturated fatty acids, *PI* peroxidizability index, *AI* atherogenicity index, *TI* thrombogenic index, *DFA* desirable fatty acids, *HFSA* hypercholesterolaemic saturated fatty acids, *h/H* hypocholesterolaemic/hypercholesterolaemic ratio.

The peroxidisability index, as one of the indices allowing to determine the shelf life of meat, differentiated both the type of muscle and the groups in terms of the birds' sex. In breast muscles, it was found to be lowest in hens' muscles, with the highest values in roosters' muscles. The pectoral muscles of capons did not differ in this respect from the other groups. The PI values for the thigh muscle were slightly different. Also here, the highest index value was recorded for the rooster's muscles, but the hen and capon thigh muscle samples were characterised by their significantly lower value, with no differences between them.

**Figure 1:**
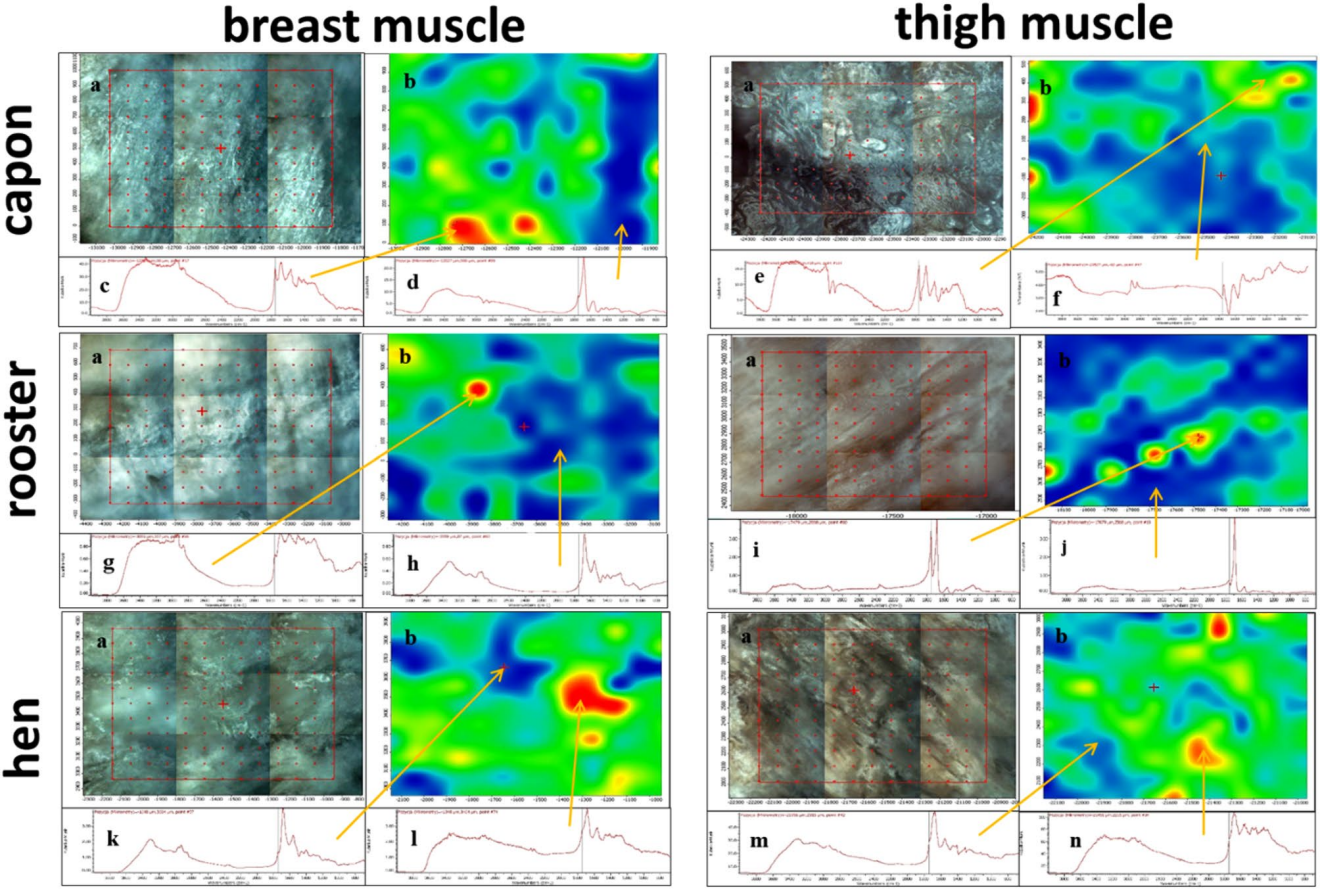
Microscopic image, chemical maps and FTIR spectra of the studied muscles. (**a**) Microscopic image with mapped area, (**b**) chemical map, (**c**) FTIR spectrum determined at 12,787 µm, 98 µm from the red area, (**d**) FTIR spectrum determined at 12,027 µm, 89 µm from the red area, (**e**) FTIR spectrum determined at position 23,327 µm, 418 µm from the red area, (**f**) FTIR spectrum determined at position 23,327 µm, 47 µm from the red area, (**g**) FTIR spectrum determined at position 3859 µm, 387 µm from the red area, (**h**) FTIR spectrum determined at position 3559 µm, 87 µm from the blue area, i) FTIR spectrum determined at position 17,479 µm, 2968 µm from the red area, (**j**) FTIR spectrum determined at position 17,679 µm, 2568 µm from the blue area, (**k**) FTIR spectrum determined at position 1648 µm, 3664 µm from the blue area, (**l**) FTIR spectrum determined at position 1348 µm, 3464 µm from the red area; (**m**) FTIR spectrum determined at position 21,956 µm, 2315 µm from the blue region, (**n**) FTIR spectrum determined at position 21,456 µm, 2215 µm from the red region.

The study showed the distribution of fatty acids in the meat samples examined (Fig. 1). The green and red areas are characterised by a higher peak intensity in the range 1744–1759 cm^−1^ corresponding to the stretching vibrations of the C=O groups from fatty acids. The blue areas show a much lower fatty acid content. The results in this range indicate the highest content of areas of high fatty acid content in hens' meat samples, with the lowest proportion of these areas in the chemical map for rooster meat, regardless of the muscle studied.

## Discussion

With the time passing after slaughter, glycolysis starts in the birds' muscles, contributing to changes in muscle pH. A number of papers^10,12^ found no effect of sex and/or caponisation on the change in muscle pH after slaughter, which is partly consistent with our own observations. Interestingly, significant differences were observed 60 min after slaughter. The pathway of glycolysis influencing post-slaughter meat traits may be influenced by a number of factors related to both genotypes^13^, nutrition or presalughter procedures^14^ which were eliminated in our study by using birds from the same flock, fed identically, therefore appears to be dependent on the sex of the birds.

Muscle colour is one of the first factors determining consumer purchasing decisions. Muscle colour has been shown to depend on a number of factors related to both the origin of the birds and the availability of pigments in their feed^15^. The age of the birds significantly forms the content of the main meat colorants, i.e. myoglobin and haem pigments, which partly explains the more intense muscle colour of all birds included in the study compared to commercial broiler chickens, similar to reports by other authors^16^. In our study, no significant differences in colour were found according to the sex of the birds. However, the results obtained by Cui et al.^10^ and Calik et al.^17^ explain better saturation of yellow colour by the increase of intramuscular fat deposition..

Normally, heat-treated poultry meat changes, due to denaturation of the haemoproteins present in the meat^18^. Remarkably, significant differences in the colour of heat-treated muscles were found in our study, even though raw meat samples did not show similar trends. Nevertheless, it should be noted that thermally treated capon breast muscles were significantly lighter in colour, which may have an impact on their consumer acceptability. Research indicates in this regard that they are more likely to choose meat with a lighter colour^19^.

In the case of drip loss, our own research has shown significant variation between groups of birds of different sexes. However, it is interesting to note that these observations are not confirmed by work comparing both capons with roosters^17,20^ and with hens or pulards^10^. In case of cooking loss the lack of statistical confirmation stated in our work may be due to the high individual variability of the trait. However, it was confirmed by the study Symeon et al.^21^ as well as Diaz et al.^22^ analysing the caponisation impact on the lying type and slow growing meat type birds, respectively. Although in the case of water holding capacity no significant differences were found between the muscle samples tested, there was a tendency for this trait to be higher in the breast muscles of cockerels compared to hens and capons, which is confirmed in the work of Calik and Obrzut^23^ where the WHC value was considerably higher in the breast muscles but also in the thigh muscles of cockerels. In our study, there were also no differences in muscle tenderness (Warner–Bratzler shear force) depending on the origin from hens, cockerels or capons. In a study by Sirri et al.^20^ such a relationship was also found but only in the case of femoral muscles (biceps femoris muscle), pectoral muscles showed less tenderness in roosters compared to capons. The differences in the results of different authors regarding muscle frailty may be due to the analysis of different bird breeds and intra-species variability.

The fact that the caponisation affects the fatty acid profile has been confirmed in many studies^6,20^. In our work, significant differences were observed for some fatty acids. For those from the MUFA group, an increase in C16:1 content was found in muscle samples, which is in line with the observations of Rikimaru et al.^24^ and Sinanoglou et al.^25^. In studies by other authors, information can also be found on the effect of surgical castration of birds on the content of several monounsaturated acids like C20:4 in their muscles^12^, which, was not confirmed in our study. The fatty acid profile was also influenced by the type of muscle studied. Some of the differences were only found in the case of femoral muscles, with significant changes in terms of PUFAs, only in the n6 group. This is partially in line with the work of Tor et al.^26^ who showed a significant interaction effect between muscle type and caponisation process in terms of the fatty acid profile including selected n6 group acids.

In addition to the observed differences in single fatty acid groups, significant differences in PI (peroxidisability index) were also found for both muscles analysed. Cocks, regardless of the muscle type, had a higher PI value than hens, and in the case of thigh muscle, the value in cocks also differed from that obtained in capons. This parameter is considered to be one of the technological parameters of meat taking into account the potential shelf life due to the autoxidation capacity of the muscle lipids^27^. The results obtained remain slightly lower than the data presented by Popova et al.^28^ for two lines of hens. The PI is a calculated value based on the proportion of individual fatty acids; it defines, in broad terms, the sensitivity of fat to oxidation and is not defined by standards. The differences in this trait observed in muscles are due to the quality and quantity of fat deposited, which is related to their physiology and motor activity. Generalizing, our results indicate better meat quality in terms of this parameter (PI) in hens and capons compared to roosters.

Fourier-transform infrared (FTIR) spectroscopy has recently become one of the analytical methods eagerly used in meat analysis due to its high accuracy and relatively low time and labour consumption. According to available reports, it has been used successfully in the detection of adulteration of raw materials used in the production of meat products^29^, but also in studies on their shelf life and spoilage processes and microbiological safety of consumers^30^. This approach is also confirmed in other works, where FTIR has been successfully used as a diagnostic tool in meat quality analysis^31,32^. In our study, the technique was used as a tool for additional verification of the chemical composition of meat obtained from sexually different groups of birds. FTIR spectroscopy images confirm observations made previously regarding the greater fatness of hens and capons carcasses relative to roosters^33^, as well as a similar relationship in MUFA levels found in this study. Thanks to the high accuracy of the method, it is therefore possible not only to indicate the quality and composition of the meat analysed, but also the distribution of nutrients in the area of the sample.

Significant differences were found concerning technological traits and levels of some fatty acids according to the sex of the birds. A considerably the lowest content of monounsaturated fatty acids was found in cocks, as well as the highest content of n3 fatty acids in capons. Regardless of the muscle type examined, meat from roosters had higher PI values. Differences in muscle composition according to the sex of the birds or the caponisation performed were also visible as changes in fat distribution in the chemical maps obtained with Fourier-transform infrared (FTIR) spectroscopy.

## Materials and methods

At the 6th week of age 120 birds (40 females and 80 males) were separated by sex based on their phenotypic visible traits and then randomly divided into 3 equal groups of 40 birds (4 replications in each) and kept in litter pens with ad libitum access to water and feed. All birds were maintained in a light programme for reproductive flocks of Greenleg Partridge. During the first week, a 24-h lighting day (40 lx) was used. Thereafter, the lighting time was reduced to 12 h (from about 2–3 to 21 weeks of birds' age) with an intensity of 5–10 lx. From 21st week the lighting was gradually increased to 16 h of daily without changing the light intensity.

40 cockerels (6 weeks old) have been surgically castrated by an authorized veterinarian. All details of caponisation procedure and the birds’ rearing and feeding were described before^33^.

Birds were kept for 24 weeks due to, according to other authors^34^ the caponization has a beneficial influence on feed intake and its conversion in light—type birds but it did not contribute to an increase in BW or the weights of major tissue components but from 20th week of age, the breast muscles proportion was higher in capons than in cockerels. The Greenleg partridge hens achieve the sexual maturity about 23–24th week of age and it is not justified to keep birds longer because of relatively low body weight gains^33^. At the age of 24 weeks, 2 birds were randomly selected from each replication subgroup (8 per group) and slaughtered in commercial poultry abattoir by decapitation (EU Regulation No. 1099/2009 of 24 September 2009 on the protection of animals at the time of slaughtering). During the dissection analysis samples of left breast muscle (*pectoralis major*) and left thigh muscles were collected.

The following meat quality features were evaluated: meat acidity (pH) 15 and 60 min, and 24 h after slaughter using pH meter CP-251; water holding capacity^35^; drip loss^36^ and cooking (thermal) loss^37^. Water holding capacity was determined by the analyse of meat and liquid spots area. The sample of 0.300 g minced meat was placed on the Whatman no 1 filter paper and then between two glass plates. The plates were pressed by 2 kg for 5 min. Drip loss was expressed as a proportion of the initial weight of meat samples to samples stored for 24 h at 4 °C. Cooking loss determined by the difference in weight between the fresh and the heat-treated sample (72 °C at the centre of the sample). After cooking cuboid cores (1 cm × 1 cm × 2 cm of edge length) were cut from the heat-treated muscles, parallel to the longitudinal orientation of the muscle fibers. Warner–Bratzler shear force was determined using a texture analyzer TA-XT plus (Stable Micro Systems Ltd. Surrey, UK) equipped with a V-shaped blade. Samples were shorn at a crosshead speed of 2 mm/s.

Color coordinates were measured twice: on freshly cut muscle surfaces and heat-treated samples using an X-Rite Color® Premiere 8200 spectrophotometer (X-Rite Inc., Michigan, USA). The thickness of the samples was at least 10 mm. The instrumental conditions were a 25.4 mm diameter area aperture. The measurement was carried out in the range of 360–740 nm. The illuminant D65 and a 10º standard colorimetric observer was used as a source of light. A white standard was used as a reference source with a specification of L* = 95.87, a* = -0.49, b* = 2.39. The results were expressed in units of the CIE LAB^38^ system, for which the distinctions reflect, respectively:

- L*—color lightness, generally adopts positive values and can take values from 0 for an extremely black body and to 100 for a perfectly white body;
- a *—chromaticity in the red-green range; means red if it is positive, green if it is negative;
- b *—yellow-blue chromaticity; means yellow if it is positive, blue if it is negative.

The change of color parameters of samples (before and after heat-treating) was calculated according to the equation and interpreted based on scale of Clydesdale^39^:

- 0 < ΔE < 1—the observer does not see any difference,
- 1 < ΔE < 2—only an experienced observer can see the difference,
- 2 < ΔE < 3,5—the difference is also noted by the non-experienced observer,
- 3,5 < ΔE < 5—the observer notes a clear color difference,
- 5 < ΔE—the observer gets the impression of two different colors.

The fatty acid profile of breast and thigh meat was analyzed using gas chromatography according to PN-EN ISO 5508:1996^40^ and PN-EN ISO 5509:2001^41^ using Varian 450-GC gas chromatograph fitted with a flame ionization detector (FID). Injector and detector temperatures were 250 °C and 300 °C, respectively. After injection, the column temperature was programmed to increase to 200 °C for 10 min, subsequently increased to 240 °C at the rate of 3 °C min^-1^. Then, the column temperature was held at the final temperature for 4 min. Helium was used as a carrier gas (3 mL min^-1^). The following indexes were calculated based on the particular fatty acid concentrations or their groups: PI—peroxidation index^42^, AI –atherogenic index and TI—thrombogenic index^43^, DFA—desirable fatty acids^44^, HSFA—hypercholesterolemic saturated fatty acids^45^ and h/H—hypocholesterolemic/ hypercholesterolemic ratio^46^.

The distribution of constituents in the pressed meat samples was examined using a Nicolet iN10 MX FTIR microscope (Thermo Scientific) equipped with a mobile XY stage using a liquid nitrogen-cooled MTC point detector. Due to the nature of the material, testing was performed using a non-destructive reflectance technique. Chemical maps were generated and processed using Omnic SpectaTM software by correlation to a selected band at position 1759 cm^−1^. This band corresponds to the stretching vibrations of the C=O groups originating from fatty acids. Prior to correlation analysis, all IR spectra were subjected to baseline correction and Kubelka–Munk correction. The resulting preparations were studied using FTIR spectroscopy. ATR spectra of the surface layer of the studied samples were recorded using a diamond crystal ATR attachment. Close contact between the ATR crystal and the surface of the test sample is a necessary condition for obtaining good-quality spectra. A Thermo Nicolet 8700 FTIR spectrometer with Smart Orbit™ diamond ATR attachment and DTGS (Deuterated Triglycine Sulphate) detector was used to record the ATR spectra. The detector guarantees signal constancy in the mid-infrared spectral range: 4000–400 cm^−1^. ATR spectra were subjected to ATR correction, baseline correction and scaled normalisation operations.

The obtained data were statistically analyzed using the SPSS 24.0 statistical package^47^. The Kolmogorov–Smirnov test was carried out to verify the normality of data. The groups were compared using ANOVA and post-hoc Tukey’s test. The significance level was defined as 5%.

### Ethics approval

The research was conducted with the approval of the Local Ethical Committee (approval no. 101/2017) at the University of Life Sciences in Lublin, Poland and all methods were performed in accordance with relevant guidelines and regulations provided by the “Directive 2010/63/EU of the European Parliament and of the Council of September 22, 2010 on the protection of animals used for scientific purposes”. The study is reported in accordance with ARRIVE guidelines (https://arriveguidelines.org). Greenleg partridge chicks consisted material of the study. Birds derived from the conservative stock maintained at Laura Kaufman Didactic and Research Station of Small Animals (University of Life Sciences in Lublin, Poland).

## Data Availability

The datasets generated and/or analyzed during the current study are available from the corresponding author on reasonable request.
